# An FBG magnetic sensor for oil flow monitoring in sandstone core

**DOI:** 10.1039/c9ra06859g

**Published:** 2019-11-04

**Authors:** Alireza Samavati, Zahra Samavati, Ahmad Fauzi Ismail, N. Yahya, M. H. D. Othman, M. A. Rahman, M. A. A. Bakar, K. N. Koo, M. F. Salebi, Iraj Sadegh Amiri

**Affiliations:** Advanced Membrane Technology Research Centre (AMTEC), Universiti Technologi Malaysia (UTM) Johor Bahru 81310 Malaysia afauzi@utm.my; Department of Fundamental and Applied Sciences, Universiti Teknologi Petronas (UTP) Bandar Seri Iskandar 32610 Malaysia; Computational Optics Research Group, Advanced Institute of Materials Science, Ton Duc Thang University Ho Chi Minh City Vietnam irajsadeghamiri@tdtu.edu.vn; Faculty of Applied Sciences, Ton Duc Thang University Ho Chi Minh City Vietnam

## Abstract

Monitoring the oil movement using a non-contact optical fiber probe during enhanced oil recovery is a novel technique to increase the efficiency of the process by distinguishing the oil position in the reservoir. A partially unclad fiber Bragg grating (FBG) coated with Fe_3_O_4_ nanoparticles as a magnetic field sensor is experimentally demonstrated. A series of six FBGs reflecting different wavelengths are fixed on the surface of sandstone. Nanofluids containing magnetite nanoparticles and alkaline-surfactant-polymer are injected continuously in two separate steps into the sandstone, which is saturated with 20% oil and 80% brine. The chamber is equipped with a solenoid that acts as a magnetic field generator. The changes in the magnetic field strength depended on the FBG-solenoid distance and the density of localized injected nanoparticles near the FBGs leads to a shift of the reflected wavelength of each single FBG accordingly. The shift is caused by the interference of different propagating modes reflected from the core-cladding and cladding-magnetite layer interfaces. The intensity of the FBG spectra decreases by injecting the nanofluid and *vice versa* for surfactant injection. The sensor response time of ∼21 s confirms the high reliability and repeatability of the sensing scheme. Movement of oil along the sandstone alters the wavelength shift in the FBG spectra.

## Abstract 2

Monitoring the oil movement using a non-contact optical fiber probe during enhanced oil recovery is a novel technique to increase the efficiency of the process by distinguishing the oil position in the reservoir.

## Graphical abstract

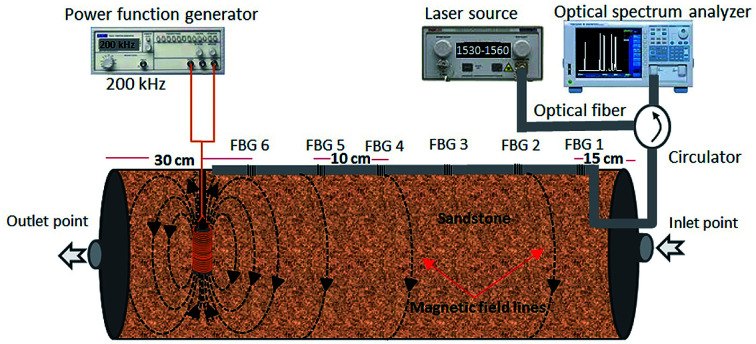

## 1. Introduction

The increase of the global demand for energy requires enhancing crude oil production. Therefore, generating the oil in an economical way is becoming more important than ever, not only to the profit-making oil corporations and nations, but also to countries, communities and individuals around the world. Advances in technology are required in key areas of the oil industry, most notably in reservoir capacity exploration and in oil-well production management, to increase yield and profit. A fundamental geometric property of a reservoir rock is porosity which is the result of its lithological, structural and compositional behavior. It can be defined as the percentage of pore volume or void space within a rock that can contain fluids. Routine core analyses generally indicate a porosity range of 10% to 25% depending on the geographic position of the reservoir. After primary and secondary recovery processes, a significant amount of oil is still trapped in the reservoir which is recovered through a tertiary process *via* nanofluid injection into the porous reservoir.^1,2^ The injected fluids in tertiary processes interact with the reservoir rock/oil system.^3–6^ These interactions might result in lower interfacial tension, oil swelling, oil viscosity reduction, wettability modification and favorable phase behavior.^7^

The increment in oil recovery using injected fluids under electromagnetic waves can be explained by different recovery mechanism as follows: (1) the dielectric polarization of nanoparticles causes deformation of oil drops shape and increase the surface area leads to more particles' adsorption, consequently reducing the interfacial tension; (2) the rate of wettability alteration increases which increase the surface free energy; (3) the improvement of mobility ratio because of electro rheological effect which increases the viscosity of nanofluids.^8^

The key to improve the oil recovery efficiency is to predict and control of oil, gas and water movement within the reservoir more accurately. Fiber Bragg Grating (FBG) is essentially a reflector created inside the core of an optical fiber. The reflector is made by permanently altering the refractive index of the core using phase mask technique. To use an FBG as a sensor, it is illuminated by a light source with a broad spectrum and the reflected wavelength shift corresponding to local measurands is monitored. Intensive development of FBG sensors have been carried out for several decades.^9^ Innovation in FBG technology extends its application in oil and gas industries, especially for health monitoring of the pipeline. FBGs offer the possibility of non-destructive, sensitive, and long-term *in situ* measurements of stress, temperature, strain, vibration and deformation in hostile environments.^10^

Usually, magnetic field sensors are fabricated using encapsulation of FBG with magnetic fluid or attachments of FBGs with magnetostrictive materials. The refractive index of nanofluid and mechanical expansion of magnetostrictive materials changes due to exposing to magnetic field. This causes shift in the reflected wavelength and make the probe acts as a magnetic field sensor.^11,12^

However, to the best of our knowledge for the first time we monitor the oil movement in porous media through detecting the external magnetic field variance using a systematic partially un-clad FBG deposited with small magnetite nanoparticle. Magnetic field changes due to moving the nanofluid along the sandstone is the sensing element which is detected by the probe located on the surface of the sandstone. Therefore, this experiment is initiated to monitor the oil movement with two injection schemes of nanofluid and surfactant. This scheme is targeted to mobilize the remaining oil in the sandstone in tertiary oil recovery process.

## 2. Materials and methods

### 2.1 FBG fabrication

The FBG was fabricated from 10 mm single-mode glass fiber optic having 8.2 μm and 125 μm core and cladding diameter respectively. The operational wavelength domains of the FBGs were chosen so that these domains did not overlap (*λ*_B_ for FBG1 = 1534 nm, FBG2 = 1544 nm, FBG3 = 1547 nm, FBG4 = 1550 nm, FBG5 = 1552 nm, and FBG6 = 1554 nm). The cladding and core material is silica doped by fluorine with refractive index of 1.4500 ± 0.0025, and silica with refractive index of 1.4765 ± 0.0025 respectively. A modulation of refractive index into the core of fiber is carried out by photo-imprints pattern which is carried out by excimer laser. The excimer laser (Lambda Physik, Germany; Model Compex 110) in the order of ±1 beam was operated at 193 nm wavelength, 50 Hz, 100 mJ pulses and pulse duration of 10 ns. The fibers were exposed to the intense UV light for duration of 300 seconds to complete the Bragg grating inscription. The period of the phase mask grating corrugation was 1072.6 nm.

### 2.2 Cladding treatment

For preparing the fiber probe, the FBG cladding needs to be partially removed and according to the penetration depth in IR region, remained cladding part should be under 2 μm thickness. From another aspect, removing process should not interrupt the total internal reflection and light propagating through the core. Therefore, to determine the appropriate optimum etching time, the transmission intensity was dynamically monitored during the immersion of the fiber in a 30% HF solution at temperature of 15 °C. The weak HF acid was used to prevent core surface corrosion that can cause a huge loss in light propagation. By monitoring the light intensity spectrum, the optimum immersing time was found to be 57 min at room temperature, and only ∼300 nm thickness of the cladding remained. This diameter was chosen because the evanescent field of light will penetrate through the FBG cladding into the surrounding medium and create light properties changes in response to external magnetic field. The rate of etching is found to be ∼1.06 μm min^−1^.

### 2.3 Coating of magnetite nanoparticles on fiber

**Fig. 1:**
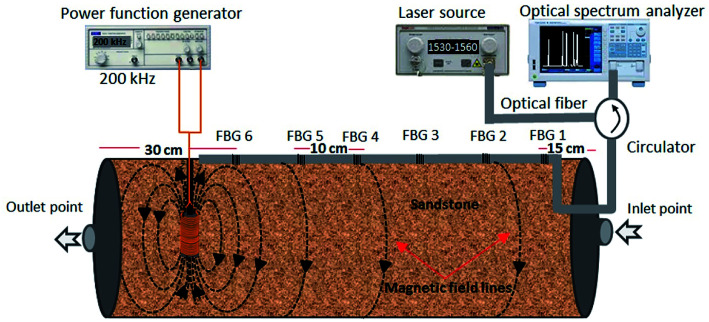
Schematic diagram of experimental setup, installation of six FBGs magnetic probe on the surface of the sandstone.

Every single partially unclad FBG was subjected for magnetite coating using polymer agent. For this purpose the Fe_3_O_4_ nanoparticles were fabricated by co-precipitating ferric chloride (FeCl_2_, Sigma Aldrich) and ferrous chloride (FeCl_3_, Qrec) under the presence of nitrogen gas. The 6.34 g of ferric chloride and 16.23 g of ferrous chloride powder were firstly dissolved in 200 mL distilled water. Chemical precipitation occurred at 30 °C under continuous stirring of solution for 30 min and adding 2 M sodium hydroxide. The reaction system was then kept at 70 °C for 3 h and pH solution ±12 at equilibrium. The black precipitate was cooled to room temperature by removing the heat source and separating by permanent magnet. The precipitate was washed with distilled water and ethanol for several times until the pH of the product reached neutral. Subsequently, the obtained precipitate was dried in the vacuum oven at 60 °C for 24 h and kept for coating. For the preparation of polymer coating agent, 20 g poly(methyl methacrylate) (PMMA) pellets purchased from Chi Mei Corporation was first dissolved in tetrahydrofuran (THF, Merck) solution with 20 wt% of PMMA. The PMMA pellets were completely dissolved in THF after stirring for 24 h at 50 °C. Then, 10 g of synthesized Fe_3_O_4_ nanoparticles were then added into the PMMA solution and stirred for 1 h and sonicated for another 1 hour to achieve uniform dispersion state. After the mixture was ready for coating, the etched FBG was dipped into the mixture and dried in the air for few minutes. The dip coating process was repeated for three times and the coated fiber was dried in the oven for 24 h at 40 °C. Low temperature treatment is necessary to completely remove the THF solvent in a slow evaporation rate as well as to enhance the adhesiveness between the nanoparticles embedded cladding and the fiber. After coating all FBGs, they are spliced to similar single mode optic fiber in series configuration by a fiber optic cutting machine (FITEL S325) and a fiber optic fusion splicer (FITEL S178) in the middle. Then the complete fiber sensor is placed in 6 mm diameter PVC tube and fixed on the surface of sandstone by epoxy glue as can be schematically seen in Fig. 1.

### 2.4 Preparation and impregnation of nanofluid

The nanoparticles were dispersed in brine as the base fluid and magnetically stirred for 1 hour to produce nanoparticles suspension. Then, the appropriate amount of sodium dodecylbenzene sulfonate (SDBS) was added to the suspensions. These suspensions were agitated in an ultrasonic bath at ambient temperature for an optimum period, to attain the weight concentration of 0.05 nanofluids. For two-step injection, firstly the sandstone was fill with fully synthetic oil (20%) and brine (80%), and then prepared nanofluids containing magnetite nanoparticles and alkaline-surfactant-polymer were injected with the rate of 1 mL min^−1^ for duration of 240 min for each.

### 2.5 Characterization of magnetite nanoparticles and probe

Field emission scanning electron microscope (FESEM, JEOLJSM 6380LA) attached with an energy dispersive X-ray spectrometer (EDX) were applied for observing the magnetite deposited layers and analyzing the elements existed in the probe structure. A broad band source with a laser wavelength ranges from 1300 nm to 1700 nm and optical spectrum analyzer (OSA) (ANDO AQ6317B) were used as a light source and detector respectively. The room temperature vibrating sample magnetometer (VSM, Lake Shore 7303-9309 VSM) was employed for determining the magnetic properties of the magnetite nanoparticle.

## 3. Results and discussion

**Fig. 2:**
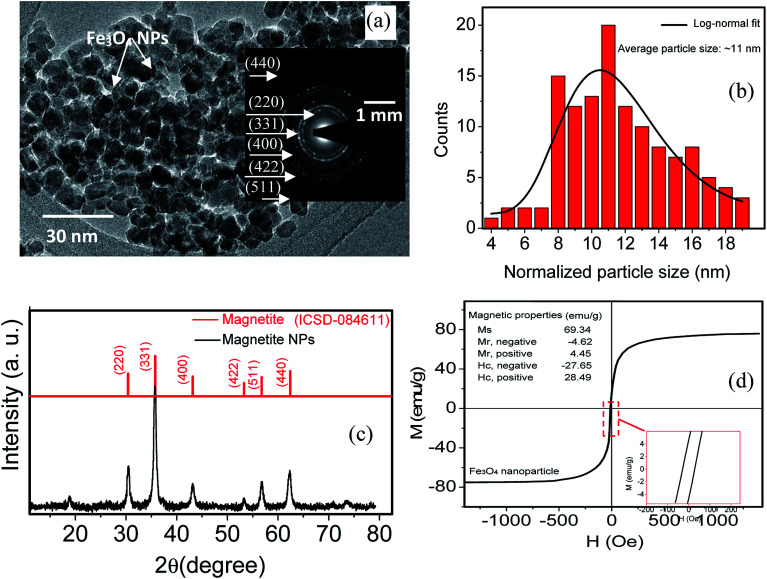
TEM image (a), size distribution (b), XRD spectra (c) and magnetic hysteresis (M–H) loops (d) of magnetite nanoparticles. Inset figure (a and c) shows SAED pattern and high magnification view of a selected area showing the small hysteresis loop, indicating a ferromagnetic component.

The structural, morphological and magnetic properties of deposited magnetite nanoparticle are analyzed and the results are depicted in Fig. 2. Fig. 2a shows TEM image of magnetite nanoparticles, clearly showing that the product is entirely composed of crystals with a relatively uniform and spherical morphology. Inset Fig. 2a shows the selected area electron diffraction (SAED) pattern recorded from an area containing a large number of nanoparticles. The rings in the SAED pattern can be indexed as the (220), (311), (400), (422), (511), and (440) reflections of the cubic magnetite which is in agreement with the XRD results.^13^ Grain size distribution was determined by measuring the mean diameter of about 130 particles on the micrographs (Fig. 2b). TEM image and bar chart indicate a uniform size distribution of magnetite nanoparticles. The average grain size of the monodisperse nanoparticles is ∼11 nm. The XRD pattern of Fe_3_O_4_ nanoparticles is shown in the Fig. 2c. The peaks at 2*θ* values of 30.1°, 35.4°, 43.1°, 53.4°, 56.9° and 62.5° are indexed as the diffractions of (220), (311), (222), (422), (511) and (440) respectively, which resembles the standard diffraction spectrum of Fe_3_O_4_ (JCPDSPDF#19-0629) with respect to its reflection peaks positions.^14^ The output of VSM measurement for magnetite nanoparticles at room temperature (Fig. 2d) indicates a hysteresis curve of magnetization with the magnetic field. Notably, a small remanent magnetization of less than 3%, which indicates a super-paramagnetic behavior, is observed. Therefore, our magnetic field sensor based on a probe coated with magnetite nanoparticles has excellent repeatability. From the hysteresis curve, the value of saturation magnetization (*M*_s_), remanent magnetization (*M*_r_), and coercivity field (*H*_c_) of the magnetite nanoparticles were determined, as shown in the inset of Fig. 2d. The magnetite nanoparticle having above features now is subjected to deposit on FBG with partially removed cladding.

**Fig. 3:**
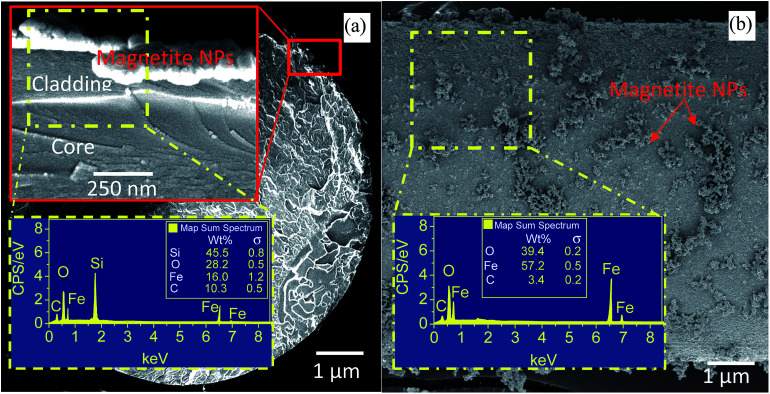
FESEM cross section (a) and surface view (b) image of coated FBG with magnetite nanoparticles. Insets show the EDX spectra of selected area.

The cross-section and top-view FESEM images of partially unclad FBG1 and the corresponding EDX spectra of selected area from coated part are depicted in Fig. 3. The magnetite layer thickness is ∼200 nm and the remain cladding thickness is ∼300 nm. For FBG2, FBG3, FBG4, FBG5 and FBG6 by using the cross section FESEM images (are not shown here) the thickness of cladding/(coated magnetite nanoparticles) are measured and the thicknesses are ∼220/(∼290), ∼200 nm/(∼320 nm), ∼190 nm/(∼320 nm), ∼210 nm/(∼300 nm) and ∼200 nm/(∼310 nm) respectively. The fiber configuration after partially removing the cladding is still in good spherical shape without appearing any disorder or deformations. The magnetite distribution on fiber surface is homogeneous however, the particle agglomeration is occurred in certain area. The EDX spectra confirm the deposition of magnetite on FBG cladding. The presence of carbon element is attributing to the tape used for fixing the sample in the EDX holder.

When total internal reflection occurred for propagating light at the core/cladding interface, the intensity is not reflected back totally at the interface and a portion of it penetrates into the cladding material. This is named evanescent wave and its intensity exponentially decays as a function of interface distance.^15^ The penetration depth (dp) of evanescent wave in the cladding material is shown by eqn (1) and it is associated to the incidence angle *θ*, RI of a core (*n*_1_), cladding (*n*_2_) and a light wavelength (*λ*).1
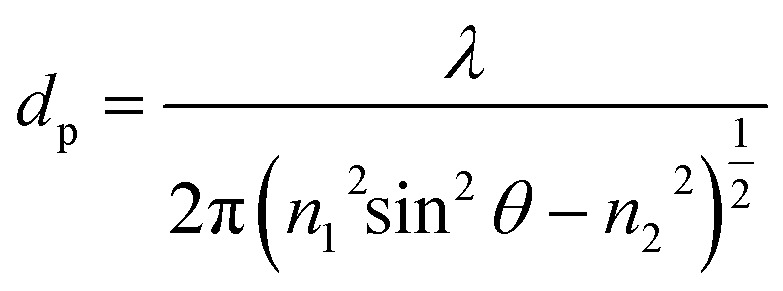


Considering the above formula, when the light wavelengths is in the range of 1530–1560 nm, the penetration depth of evanescent wave is ∼2 μm, which confirms that removing a major part of FBG fiber cladding until ∼300 nm (Fig. 3a) can allow evanescent wave reach the magnetite nanoparticles.

**Fig. 4:**
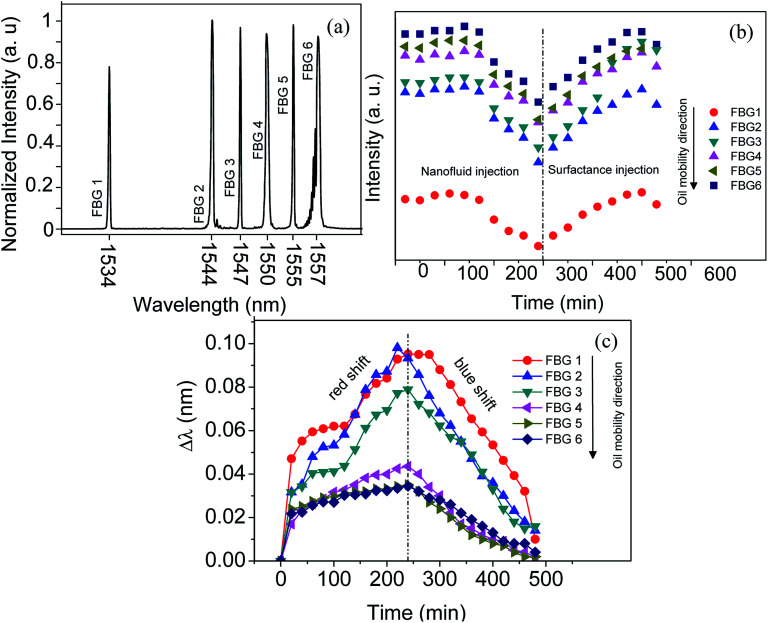
The reflection spectra of six FBGs fixed along the sandstone (a). The intensity modulation during nanofluid and surfactant injection (b), wavelength shift as a function of injection time (c).

The Reflection spectra from six FBGs probe and their corresponding intensity and wavelength modulation as a function of injection time for entire filling process including 240 min magnetite nanofluid injection continued by 240 min surfactant injection is illustrated in Fig. 4. The intensity and wavelength of FBGs are recorded every 30 and 20 min respectively. When super paramagnetic nanoparticles are in the presence of an external magnetic field, they act as nano-magnets and, accordingly, exhibit several useful properties such as moving in controlled direction, easily detection, generating highly localized intense heat and completely losing their magnetism instantly once the external magnetic field is removed.

Under the magnetic field, generally there are two forces acting on the magnetic nanoparticles in ferrofluid: (1) the magnetic force (*F*_m_) generated by the applied magnetic field gradient, and (2) the fluidic force (*F*_f_) exerted by the suspending medium on a moving magnetic nanoparticles. Other forces, such as buoyancy, the gravitational force, and the interaction between particles, can be neglected for magnetic nanopareticles in the fluid.^16^ The magnetic force on the particles is usually derived by the following formula:^17^

2 *F⃑*_m_ = *μ*_0_*V*_m_*χ*(*H⃑*_a_∇)*H⃑*_a_

where *μ*_0_ is the magnetic permeability of free space, *V*_m_ is the volume of the particle, *χ* is the magnetic susceptibility, and *H*_a_ is the applied external magnetic field. The fluidic force for a spherical particle is determined by Stokes' law:

3 *F⃑*_f_ = −6π*ηr*_p_(*v⃑*_p_ − *u⃑*)

where *r*_p_ and *V*_p_ are the hydrodynamic radius and velocity of the particle and *η* and *u* are the viscosity and velocity of the fluid, respectively. Since the magnetic susceptibility of magnetic nanoparticles was measured based on the total magnetic particle, the magnetic radius in eqn (2) and the hydrodynamic radius in eqn (3) can be considered equal. The total force on magnetic nanofluid in the magnetic field and viscous fluid can be written as bellow:

4 *F*_total_ = *μ*_0_*V*_m_*χ*(*H⃑*_a_∇)*H⃑*_a_ − 6π*ηr*_p_*v⃑*_p_

The fluid has an static condition in the experiment therefore, the velocity of the fluid *u* can be set as zero.

**Fig. 5:**
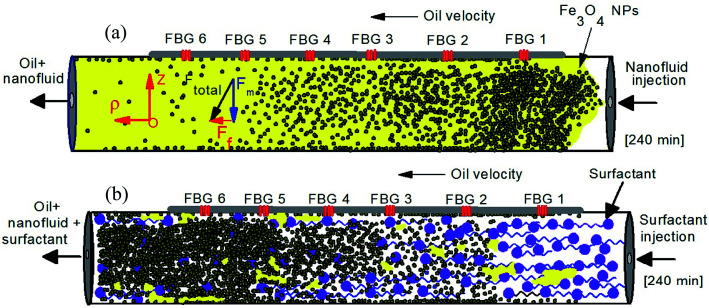
Schematic mechanism of injecting the magnetite nanofluid (a) and surfactant (b) for enhance oil recovery process.

When the magnetite nanoparticles move under the sum of these two forces as can be seen in the schematic diagram in Fig. 5a, the total force direction lead the particles move slowly and push them to the surface of sandstone. Therefore, low velocity and continuing injection of nanofluid together cause to increase the density of magnetic nanoparticles closer to the injection point. The number of density gradually decreases along the sandstone by increasing the distance from injection point as can be seen in schematic Fig. 5a.

**Fig. 6:**
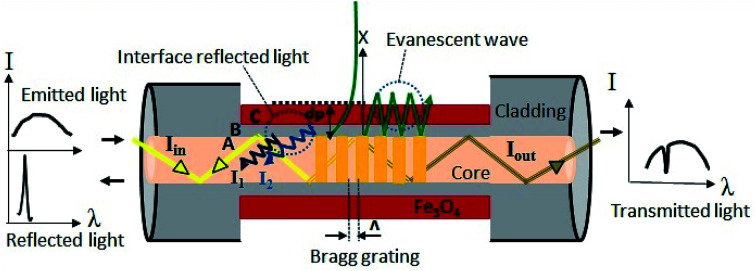
The sensing mechanism based on interferometer of core and cladding mode of FBG coated with magnetite nanoparticles.

In the case of paramagnetic materials, magnetism arises once the magnetic field introduce because one of the most fundamental properties of electron is that it acts as a magnetic dipole (analogous to a small current carrying loop). As a result, the electrons also have an associated magnetic dipole moment (intrinsic and orbital). These magnetic dipoles are randomly oriented, so that the net magnetic moment for the material is zero. However, when an external magnetic field is introduced, these dipoles align themselves along the direction of the external magnetic field, thus the total magnetic field inside the material increases.

External magnetic field can tune the refractive index of magnetite nanoparticles. In the first-order approximation, the dielectric constant is linearly proportional to the magnetization. In the dielectric tensor *ε* (*M*, *ω*), only *ε*_*xy*_ pair of diagonal elements is non-zero, when *M* is in the *z* direction. The *ε*_*xy*_ components in the first-order of magnetization vary linearly with M and increase the magneto optical effects. The refractive indices depend on magnetization and can be expressed by following equation:^18–21^

5 *n*^2^ = *aM* + *b*

6 *M* = *χ*_V(magnetite)_*H*

where *χ*_V(magnetite)_ is called the volume magnetic susceptibility for magnetite where *a* and *b* represent the proportional constants and *M* is the magnetization. It can be concluded that exposing the coated magnetite nanoparticles to external magnetic field causes magnetization of the ferromagnetic materials, by changing the magnetization at different external magnetic fields explained above the refractive index of the magnetite nanoparticles is varied. Stronger magnetic field leads to higher refractive index. When the incident light is reflected from the Bragg grating, a part of main mode light in the core escapes into cladding layer which known as evanescent wave and motivate cladding modes. The transmission angles for the majority of light rays inside cladding are still greater than the corresponding critical angles that consequences in not satisfying the total reflection condition. As a result majority of cladding mode light are vanished in the surrounding environment. Furthermore, the absorption of light in the coated medium can be determined by imaginary part of the refractive index which is shown by following equation,^18^7
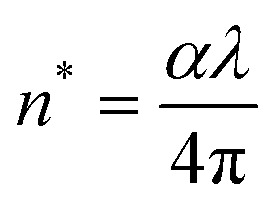
where *λ* is the propagating light wavelength and *α* is constant. Therefore, higher refractive index media leads to higher absorption of evanescent wave and decreasing the reflected light intensity by FBGs. Thus, as can be seen in the Fig. 4b the intensity of reflected light for all FBGs decreases by injecting the nanofluid. However, when the surfactant is injected the reverse mechanism is occurred and by pushing the nanofluids and remain oil by surfactant the density of nanofluids become higher near the outlet point (Fig. 5b). Therefore, this reverse mechanism increases the intensity gradually. Moreover, the intensity for FBG closer to injection point is lower than the far one, which is in good agreement with above theory.

The square of the effective refractive index varies linearly with the magnetic strength. The above facts show a strong correlation among the magnetization, the refractive index, and the characteristics of the ferromagnetic coated nanoparticles, which provides firm evidence to support our proposed mechanism for the observation of magnetically modulated propagating wavelength through refractive index changes of the magnetite in next section.

Fig. 4c illustrates the wavelength shift for all FBGs as a function of injection time (nanofluid and surfactant). The overall trend of wavelength shift is increased over time for all FBGs during nanofluid injection. The higher wavelength shift is occurred for FBG1. Going from FBG1 to FBG6 the wavelength shift is decreased. The amount of Δ*λ* is proportional to the nanofluid density. By injecting the surfactant the wavelengths are blue shifted and reverse trend occurred compare to nanofluid injection. On the other word wavelength shift is corresponded to movement of nanofluid (oil movement). Therefore, it can be conclude that the distribution of nanofluid (oil) volumetrically can be determined by such method. The mechanism for wavelength shift is explained as bellow.

When the light encounters in the two reflective parallel surfaces, for every internal reflection the portion of it is reflected back and the remain is transmitted out. The multiple beams interference is occurred once these beams are bounded. In our experimental setup the two interfaces are core-cladding and cladding-magnetite thin film. After recombination, the difference optical path length of these two spectrums that reflected from two different interfaces leads to occurrence of constructive and destructive interference depending on the phase variance. The interference spectrum is determined by the following two-beam interference equation as follows:^22^8
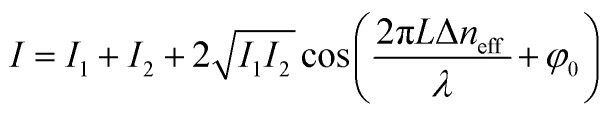
Here *I* is the intensity of total interference signal detected by spectral analyzer. *I*_1_ and *I*_2_ are the intensities of core mode and cladding modes reflected light respectively, which are shown in schematic diagram in Fig. 6. *φ*_0_ is initial phase difference, *L* is the length of sensing part and *λ* is the wavelength. Δ*n*_eff_ is the difference between effective RI of core mode and cladding modes which is explained as following:

9 Δ*n*_eff_ = *n*^core^_eff_ − *n*^clad^_eff_

It can be seen from eqn (7) that the max transmission is taking place when10
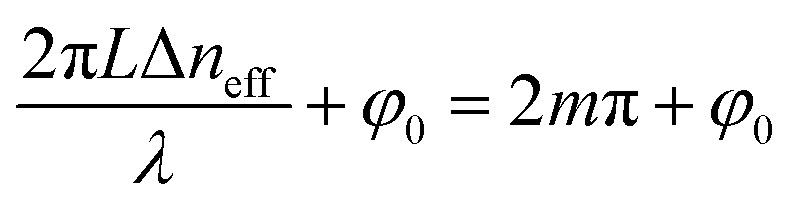
and *m* is an integer number. Therefore, the transmission spectrum reveals dips at following wavelengths:11
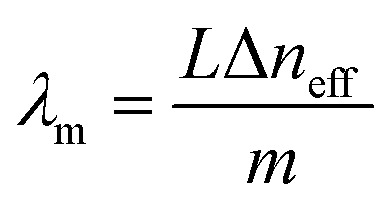


The changes in the external magnetic field do not affect the *n*^core^_eff_ and it is almost unchanged, however, as it is mentioned in eqn (8) the *n*^clad^_eff_ varies once the external magnetic field strength changes. Therefore, any small change in the external magnetic field caused by different variables such as distance, medium and power frequency leads to altering the *n*^clad^_eff_ and Δ*n*_eff_. Therefore, the dip wavelength shift Δ*λ*_m_ can be written as:12
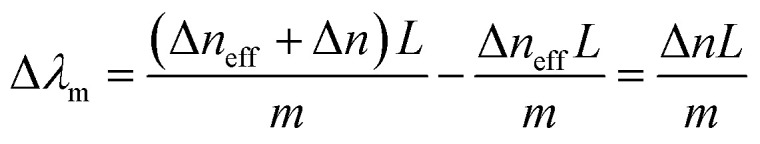
where Δ*n* is the difference between effective RI of two states corresponding to different magnetic field strengths. Considering eqn (11), it can be concluded that the varying of sensing length *L* and RI difference Δ*n* can both result in detected light wavelength shift. When the *L* keeps constant (15 mm), the wavelength shift is the linear function of RI changes. The changing of refractive index of coated magnetite nanoparticle for every FBGs along the chamber is explained in previous section. Therefore, considering all above phenomena and mechanisms any changes in the magnetic field strength propagated along the chamber can finally modify the RI of the magnetite thin layer and consequently, leads to wavelength shift.

**Fig. 7:**
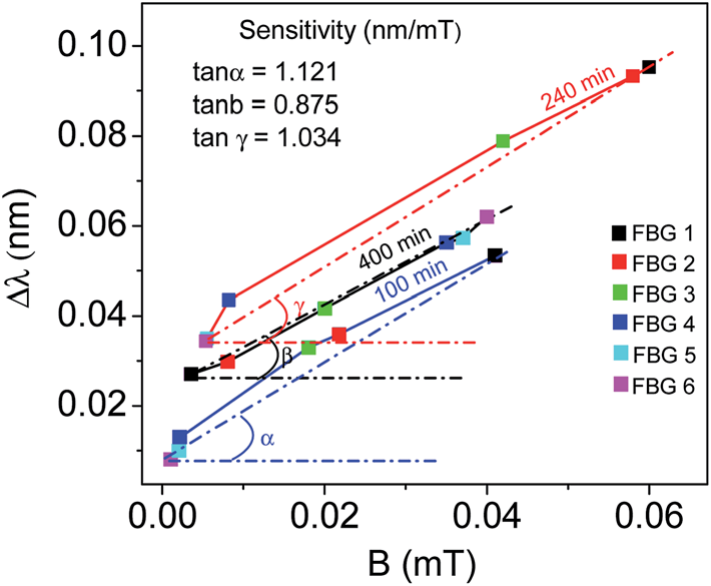
FBG spectrum shift as a function of magnetic field after 100, 240 and 400 min of nanofluid and surfactant injection.

Fig. 7 shows the wavelength shift corresponding to magnetic field strength which is experimentally calculated using tesla meter at the FBGs positions after injection of nanofluid (100 min, 240 min) and surfactant (400 min). The average sensitivity of the FBG probe is measured using the slope of the plots and listed in Table 1. The sensitivity of the probe depends on not only the manufacturing process (for instance, the accuracy of the removing process for cladding and the homogeneous coating of small magnetite nanoparticle on the partially unclad part), but also the magnetic field intensity received by the FBG probe. For higher magnetic field, the sensitivity is higher, and *vice versa*. It should be mentioned that the same condition (uncladding and coating) in manufacturing process for all the FBGs are considered. Therefore, different sensitivity may originated from exposing to different magnetic field strength. From the M–H hysteresis loop shown in Fig. 4, the saturation magnetization of magnetite nanoparticle is ∼69 emu g^−1^, and its absolute coercive force is ∼28 Oe. This value is still beyond the magnetization saturation threshold, and therefore, the sensitivity of the probe, which arises from changes in the magnetization of the nanoparticles, is a function of the external applied magnetic field. From Table 1, it is seen that the sensors have higher sensitivity compare to the sensors in previous works.

**Table 1:** Performance of FBG sensors in terms of magnetic field detection

Detection mechanism	Ferrofluid/nanoparticle	Detection range	Sensitivity	Ref.
Wavelength shift	Fe_3_O_4_	0–25 mT	86 pm/25 mT (no linear behavior)	23
Intensity of reflected power	EMG 605	0–14 mT	1470 nW mT^−1^	24
Wavelength shift	EMG 705	0–32 mT	106 pm/35 mT (no linear behavior)	25
Wavelength shift	Fe_3_O_4_	0–166 mT	7400 pm/166 mT (no linear behavior)	26
Cladding mode intensity	Ferromagnetic particles	7–15 mT	−0.78 dB mT^−1^	27
Wavelength shift	Fe_3_O_4_ < 10 nm	0–0.06 mT	1009 pm mT^−1^	Current study

**Fig. 8:**
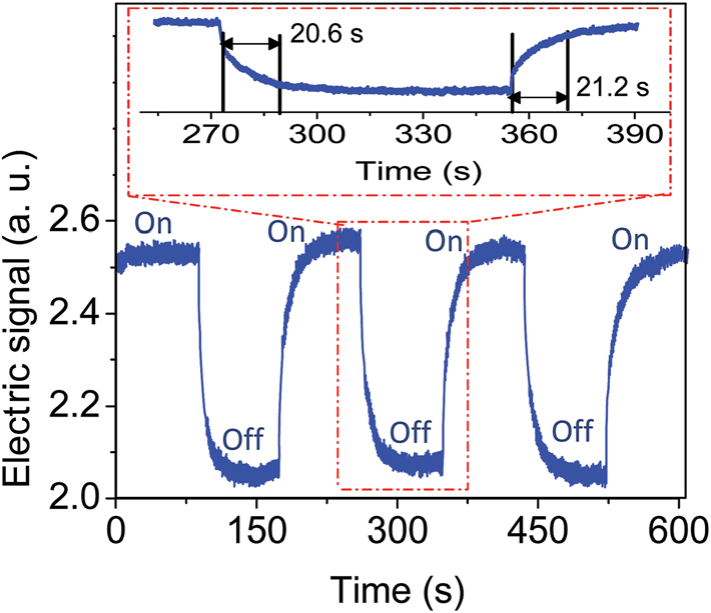
Time response plot of the fabricated FBG magnetic field sensor under on–off condition. The range of magnetic field is from 0 to 100 Oe.

The great importance for practical applications of magnetic sensor is time response and repeatability. Using on–off control of the magnetic field from 0 to 100 Oe a few circles of time response test was carried out, to determine the response time and the repeatability of the FBG probes. Three circles of temporal change of the reflection power reshown in Fig. 8. It can be seen that when the electromagnet turned off, the signal recovered to the original level. A rise and fall time according to 10% baseline to 90% signal maximum are determined as 21.2 s and 20.6 s respectively which is depicted in enlarged cycle in Fig. 8. The response time of the sensor mainly depends on the external magnetic field strength. The identical response behavior of the sensor confirms the high reliability and repeatability of the sensing scheme.

## 4. Conclusion

A series of six FBGs partially unclad, coated with magnetite and fix on the surface of sandstone without direct contacting with the stone which saturated by 20% oil and 80% saline. Enhanced oil recovery is promoted by injecting the magnetite nanofluid following by surfactant in the sandstone at the presence of magnetic field generated by solenoid located near the outlet point. Moving the oil in the sandstone is monitored by changing the sensing elements of FBG's including intensity and wavelength. The intensity decreases by injecting the nanofluid and increases by injecting the surfactant. The wavelength is red-shifted once the nanofluid injected and blue-shifted after injection of surfactant. The sensor response time is ∼21 s which approves the high reliability and repeatability of the probes. The wavelength shift is increased by moving the oil along the sandstone. The novel design of our experiment along with accurate fabrication of highly sensitive FBG probes may give insight into their use in the oil and gas industry.

## Conflicts of interest

There is no conflicts to declare.

## Supplementary Material
